# Acquisition, maintenance and adaptation of invasion inhibitory antibodies against *Plasmodium falciparum* invasion ligands involved in immune evasion

**DOI:** 10.1371/journal.pone.0182187

**Published:** 2017-08-07

**Authors:** Muyideen K. Tijani, Oluwatoyin A. Babalola, Alex B. Odaibo, Chiaka I. Anumudu, Adanze O. Asinobi, Olajumoke A. Morenikeji, Michael C. Asuzu, Christine Langer, Linda Reiling, James G. Beeson, Mats Wahlgren, Roseangela I. Nwuba, Kristina E. M. Persson

**Affiliations:** 1 Cellular Parasitology Programme, Cell Biology and Genetics Unit, Department of Zoology, University of Ibadan, Ibadan, Nigeria; 2 Department of Microbiology, Tumor and Cell Biology (MTC), Karolinska Institutet, Stockholm, Sweden; 3 Department of Paediatrics, University College Hospital, University of Ibadan, Ibadan, Nigeria; 4 Department of Preventive Medicine and Primary Care, College of Medicine, University of Ibadan, Ibadan, Nigeria; 5 The Macfarlane Burnet Institute for Medical Research and Public Health, Melbourne, Victoria, Australia; 6 Department of Laboratory Medicine, University Hospital, Lund University, Lund, Sweden; Université Pierre et Marie Curie, FRANCE

## Abstract

Erythrocyte-binding antigens (EBAs) and *P*. *falciparum* reticulocyte-binding homologue proteins (PfRhs) are two important protein families that can vary in expression and utilization by *P*. *falciparum* to evade inhibitory antibodies. We evaluated antibodies at repeated time-points among individuals living in an endemic region in Nigeria over almost one year against these vaccine candidates. Antibody levels against EBA140, EBA175, EBA181, PfRh2, PfRh4, and MSP2, were measured by ELISA. We also used parasites with disrupted EBA140, EBA175 and EBA181 genes to show that all these were targets of invasion inhibitory antibodies. However, antigenic targets of inhibitory antibodies were not stable and changed substantially over time in most individuals, independent of age. Antibodies levels measured by ELISA also varied within and between individuals over time and the antibodies against EBA181, PfRh2 and MSP2 declined more rapidly in younger individuals (≤15 years) compared with older (>15). The breadth of high antibody responses over time was more influenced by age than by the frequency of infection. High antibody levels were associated with a more stable invasion inhibitory response, which could indicate that during the long process of formation of immunity, many changes not only in levels but also in functional responses are needed. This is an important finding in understanding natural immunity against malaria, which is essential for making an efficacious vaccine.

## Introduction

An estimated 3.4 billion people are at risk of malaria worldwide and about 429,000 deaths due to malaria were reported in the year 2015 alone [1]. Unfortunately, there is not yet a highly efficacious vaccine for malaria and the final results from the phase 3 trial of the most advanced malaria vaccine, RTS,S, showed only modest efficacy [2]. Therefore, there is need for better understanding of naturally acquired immunity against malaria to be able to develop effective vaccines. Classical studies in the 1960’s in which passively transferred antibodies alleviated symptoms of malaria in sick children form the basis of the idea that vaccines based on asexual stage antigens could be successful [3,4].

Merozoite proteins that mediate erythrocyte invasion are important targets of protective antibodies in naturally exposed individuals [5,6] and such antibodies can either inhibit erythrocyte invasion directly [7], recruit complement to inhibit invasion and lyse merozoites [8] or function by antibody-mediated cellular mechanisms such as opsonic phagocytosis [6]. However, naturally acquired immunity is not sterile and so the parasite is able to infect immune individuals living in endemic areas repeatedly, albeit with less severe consequences [9]. *P*. *falciparum* can cause repeated infections over time and evade evolving immune responses. Mechanisms through which *P*. *falciparum* is able to cause repeated infections appear to be at least partly mediated by its ability to vary invasion ligand expression and use, as well as through antigenic diversity of key immune targets [10]. Previous studies suggest that phenotypic variation based on selective expression of members of the erythrocyte-binding antigen (EBAs) and *P*. *falciparum* reticulocyte-binding homologue (PfRhs) invasion ligands families is an important immune evasion mechanism utilized by the parasite [11].

EBAs and PfRhs mediate important steps in erythrocyte invasion [12] and are notable for their variation in expression and/or utilization in invasion that results in the ability of *P*. *falciparum* to use different invasion pathways. Significant diversity in the expression of EBAs and PfRh ligands has been well described in clinical isolates [13], and appears to represent a mechanism that facilitates the parasites ability to adapt to variability in host erythrocyte surface receptors and to evade the immune responses directed against them [10,11,14]. The EBA proteins are sequestered in the microneme and consist of EBA140 [15], EBA175 [16], EBA181 [17] and EBL 1 [18]. The erythrocyte receptors for EBA175, EBA140 and EBL 1 are known to be glycophorin A [19,20], C [21], and B [22], respectively. The definitive receptor for EBA181 is not known, but it binds to sialic acid (SA) on erythrocytes and to band 4.1 protein [17,23]. The PfRh family of proteins are stored in the rhoptry and consist of PfRh1, PfRh2a, PfRh2b, PfRh4 and PfRh5 (reviewed in 11). Except for PfRh4 and PfRh5 where the receptors have been shown to be complement receptor 1 (CR1) [24], and basigin (CD137) [25], respectively, the receptors of the remaining PfRh proteins are still unknown. PfRh2a and PfRh2b have about 80% homology covering mainly the N terminal part of the protein. Other members of these families of invasion ligands, PfRh3 and EBA165, appear to be pseudogenes in all the *P*. *falciparum* strains that have been studied so far [26]. EBA and PfRh proteins are mediators of major invasion pathways utilized by *P*. *falciparum*, and they can be divided into SA-dependent and SA-independent pathways. EBA 140, EBA175, EBA181 and PfRh1 are the main mediators of the SA-dependent pathway [17] while PfRh2a, PfRh2b and PfRh4 are involved in the SA-independent pathway [27], but a role has also been described for PfRh2 in the SA-dependent pathway.

EBAs and PfRhs elicit antibody responses in individuals living in malaria endemic regions [14,28,29]. A recent study has shown that antibodies to various regions of the EBAs and PfRh ligands are associated with protective immunity [5]. Due to their widespread immunogenicity and functional roles in erythrocyte invasion, EBA and PfRh ligands are promising vaccine candidates. Vaccines based on the most studied of the EBAs, EBA175 (Region II, RII), have been tested singly or in combination with other malaria parasite recombinant proteins in phase 1 clinical trials [30,31]. Meanwhile, a vaccine composed of RIII-V of EBA175 has been shown to elicit highly functional invasion inhibitory antibodies in rabbits better than EBA175 RII [32].

In this study we aimed to understand the development of acquired antibody responses to EBA and PfRh proteins and invasion pathways, their potential importance in immune evasion, and how these responses are maintained or adapt over time. We evaluated antibody responses to EBA140, EBA175, EBA181 (regions III-V for all EBAs), PfRh2, PfRh4 and MSP2 by ELISA, and invasion inhibitory assays were performed using wild-type parasites and parasites with genetically disrupted EBA140, EBA175 or EBA181 ligands. Blood samples from a longitudinal study were used, spanning over a time period of almost one year, giving a unique opportunity to longitudinally follow the variation and adaptation in levels and function of antibodies directed against multiple invasion ligands of *P*. *falciparum*. This work provides an important contribution in understanding how individuals adapt to merozoite invasion diversity and acquire protective immunity, and why it has been difficult to maintain vaccine efficacies over longer time periods in vaccine trials that have been conducted to date.

## Materials and methods

### Study area and participants

This study was conducted in Igbo-Ora, a rural town in Ibarapa North local government area of Oyo state, Nigeria. It is located at about 80 kilometers west of Ibadan, the capital of Oyo state. Malaria transmission is seasonal and high during the rainy season, April to October, and low in the dry season, which runs from November to March. Full description of the study site has been published earlier [33,34]. From July, 2009 to June, 2010 306 individuals aged 5 to 70 years were enrolled for an 8 months longitudinal study. The enrollment exclusion criteria were signs of severe hepatic or renal dysfunction, sickle cell disease (HbSS and HbSC), deficiency in Glucose-6-phosphate dehydrogenase and seropositivity for HIV. Venous blood samples (5 mL) were collected monthly and additional samples were collected when the participants developed malaria, and plasma samples obtained were stored at -80°C. 156 consistent individuals (participants of at least six months) were included in the final longitudinal analysis and they contributed a total of 1,134 samples. All participants that developed uncomplicated malaria during the study period were treated according to the WHO recommendation: oral artesunate/lumefantrine for five days. The case definition for malaria was an axillary temperature of ≥37.5°C, *P*. *falciparum* asexual stage parasitaemia of ≥5,000 parasites/μL, and no signs of other diseases. Self-reported cases of malaria were also considered. No cases of severe malaria were encountered throughout the study period.

### Ethical issues

Written informed consent was obtained from adult participants or parent/legal guardians for the children. Ethical approval for this study was granted by the University of Ibadan and University College Hospital, Ibadan, Ethical Committee (UI/IRC/06/0038) and Stockholm Ethical review board (2013/4:8).

### Parasitaemia determination

Thick and thin blood smears collected monthly were stained with Giemsa and counted against 500 leucocytes. The parasite density was expressed as the number of asexual *P*. *falciparum* parasites/μL microliter of blood assuming 8000 leucocytes/μL. A smear was considered negative when no parasites were observed after counting microscopic fields that included at least 500 leucocytes [35].

### Blood genotype

Routine haemoglobin electrophoresis (HBE) using cellulose acetate paper in alkaline buffer (pH 8.6) was used to determine the genotype of all blood samples.

### Parasite antigens

Most of the recombinant proteins used in this study have been described earlier and they were all expressed in *Escherichia coli* [28,36–38]: 3D7 EBA140 region III-V (aa 746–1045), 3D7 EBA175 region III-V (aa 761–1271), 3D7 EBA181 region III-V (aa 755–1339), PfRh2 (aa 2027–2533), 3D7 PfRh4 (aa 1160–1370) and FC27 MSP2 (full length protein).

### Enzyme-linked Immunosorbent Assay (ELISA)

Antibodies to recombinant proteins were measured by ELISA using well established methods [28]. Maxisorp microtiter plates (Nunc, Roskilde, Denmark) were coated with 1μg/mL antigen in PBS at 4°C overnight, washed ×3 in PBS/0.05% Tween 20, blocked with 10% skimmed milk/PBS/0.05%Tween 20, washed ×3 in PBS-Tween 20, plasma samples at 1:50 for EBAs/PfRhs or 1:100 for MSP2 in 5% skimmed milk/PBS-Tween 20 were added in duplicates. Plates were incubated at 37°C for 2 hours, washed ×3, incubated 1 hour at 37°C with horseradish peroxidase-conjugated goat anti-human IgG (Sigma, Germany) at 1/1000 dilution in 5% skimmed milk/PBS-Tween 20 0.05%, washed ×3 in PBS/0.05%Tween 20, washed ×3 in PBS/0.05% Tween 20 and then azino-bis-3-ethylbenthiazoline-6-sulphurnic acid, ABTS, was added. Colour was developed in the dark at room temperature for 30–40 minutes and the absorbance read at 405 nm. For each plasma sample, the absorbance from wells containing GST only was subtracted from the absorbance from the EBA and PfRhs GST fusion proteins. This was because all recombinant proteins used, except MSP2, were expressed as GST fusion proteins; therefore GST fusion tag was expressed on its own and used as a negative control antigen. A pool of immune Ugandan samples and a Nigerian sample were used as positive controls and two Swedish unexposed samples were used as negative controls on all plates to enable standardization. A plasma sample was considered positive when absorbance was higher than the mean + 3 standard deviations of 12 Swedish malaria naïve samples obtained from the Karolinska Hospital Blood Bank. A complete set of longitudinal samples from each individual was assayed on the same plate throughout.

### *Plasmodium falciparum* culture and invasion assay

Methods for measuring invasion inhibitory antibodies in plasma have been evaluated in details elsewhere [14,39]. *P*. *falciparum* lines 3D7WT, 3D7EBA140KO, 3D7EBA175KO, and 3D7EBA181KO were cultured in vitro in 25 mL culture flasks (Nunc, Denmark) using human group O^+^ erythrocytes at about 4% hematocrit in RPMI-HEPES supplement with 25 mM NaHCO3, 25 μg/mL of gentamicin, 5 mM L-glutamine 5% (v/v) heat-inactivated pooled human sera (AB+ blood group) from donors resident in Sweden. The cultures were maintained in candle boxes and synchronized twice a week by suspending the culture pellet in 5% D-sorbitol (Sigma, Germany) until used for invasion assays. The parasite strains were kind gifts from Professor Alan Cowman, Australia.

All plasma samples used in the invasion assays were dialysed to remove nonspecific inhibitory activities as described earlier [39]. Briefly, 150 μL of plasma samples were dialysed against PBS (pH 7.4) using 50-kDa MWCO microdialysis tubes (G-Biosciences; MO) and then reconstituted to the starting volume using 100-kDa MWCO (PALL life Sciences; MI) centrifugal concentration tubes. Invasion assays were initiated with all parasite strains at the late trophozoite stage with starting parasitaemia of 0.3–0.5% at 2% hematocrit in U-bottom 96-well plates (culture volume of 50μL per well). Each well contained 45 μL of culture plus 5 μL test sample, all samples were tested in duplicate. After approximately 48 hours, 8–10 μL of culture medium was gently mixed into each well. The parasitaemia was measured by flow cytometry (FASCAN; BD) at the end of the assay (after ~ 90 hours) after staining with acridine orange (10 μg/mL) and fixing the cells with 20% / 2% formaldehyde/ glutaraldehyde. Flow cytometry data was evaluated using Flowjo software (Tree Star, Inc.). A 10% difference in invasion between the different parasite lines was used as the cutoff for differential inhibition by plasma samples. Three individuals did not have sufficient plasma for invasion assays so samples from153 individuals were used here.

### Statistical analyses

Statistical analyses were carried out using GraphPad Prism V5.0 software, SPSS V20 and STATA 12. ANOVA or Kruskal-Wallis with Dunn’s multiple comparison post-hoc tests were used to examine differences in antibody levels between ordered categorical variables (months; none, low and high exposure groups; invasion inhibition reaction patterns). Chi square or Fisher exact test was used to compare proportions between different categories. Mann Whitney test was used to compare antibody levels between two groups. Survival analysis of antibody levels equal or below 50% of the day 0 was examined using Kaplan-Meier plots and log rank test. Correlation between two continuous variables was determined by Spearman’s rank correlation. The formation of clusters based on antibody response selectivity was determined using agglomerative hierarchical clustering. Multivariate regression analysis was used to test the association between breadth of high antibody response, age, median parasitemia and sex.

#### Definitions

Individuals with median parasitaemias (median of 8 months) equal to zero were considered as having low frequency parasitaemia (n = 71, parasites not detected in 4 throughout) while individuals with median parasitaemias of greater than zero were regarded as the high frequency parasitaemia group (n = 85). The overall examination of the data showed that median parasitaemia is more representative of the spread of parasitaemia than mean parasitaemia. Individuals that consistently produced antibody levels equal to or greater than the 75^th^ percentile value of the total antibody levels measure by ELISA were regarded as high responders.

## Results

### *P*. *falciparum* parasitaemia in the study population over time

Parasitaemia of individuals (156) that participated in this study was evaluated microscopically at every point of sample collection. *P*. *falciparum* was detected at one time or the other in a majority of the participants (97.5%), except for 4 children that consistently had *P*. *falciparum*-negative slides throughout the study period. Mean monthly parasitaemia increased from July (299 parasites/μl of blood), which coincided with the early part of the rainy season (April to October), and peaked in the month of October (537 parasites/μl of blood) (S1 Fig). Pairwise comparison of the monthly parasitaemia showed significant differences (p<0.05) for most months, but the mean parasitaemia for the rainy season months (343 parasites/μl of blood) was not significantly higher than the dry season months (224 parasites/μl of blood). Also, pairwise comparison of parasite prevalence (frequency of *P*. *falciparum*-positive slides at any time point) showed significant differences only between February and all the months except August. January vs March (p = 0.02) and January vs May (p = 0.02) were also significantly different in parasite prevalence (S1 Fig). Average prevalence for the rainy season (2009) was 49.6% while the average prevalence for the dry season (November-March, 2009/2010) was 48.5%. There was no statistical difference between parasite prevalence between the two seasons.

### IgG antibody responses to merozoite antigens measured by ELISA

**Fig 1:**
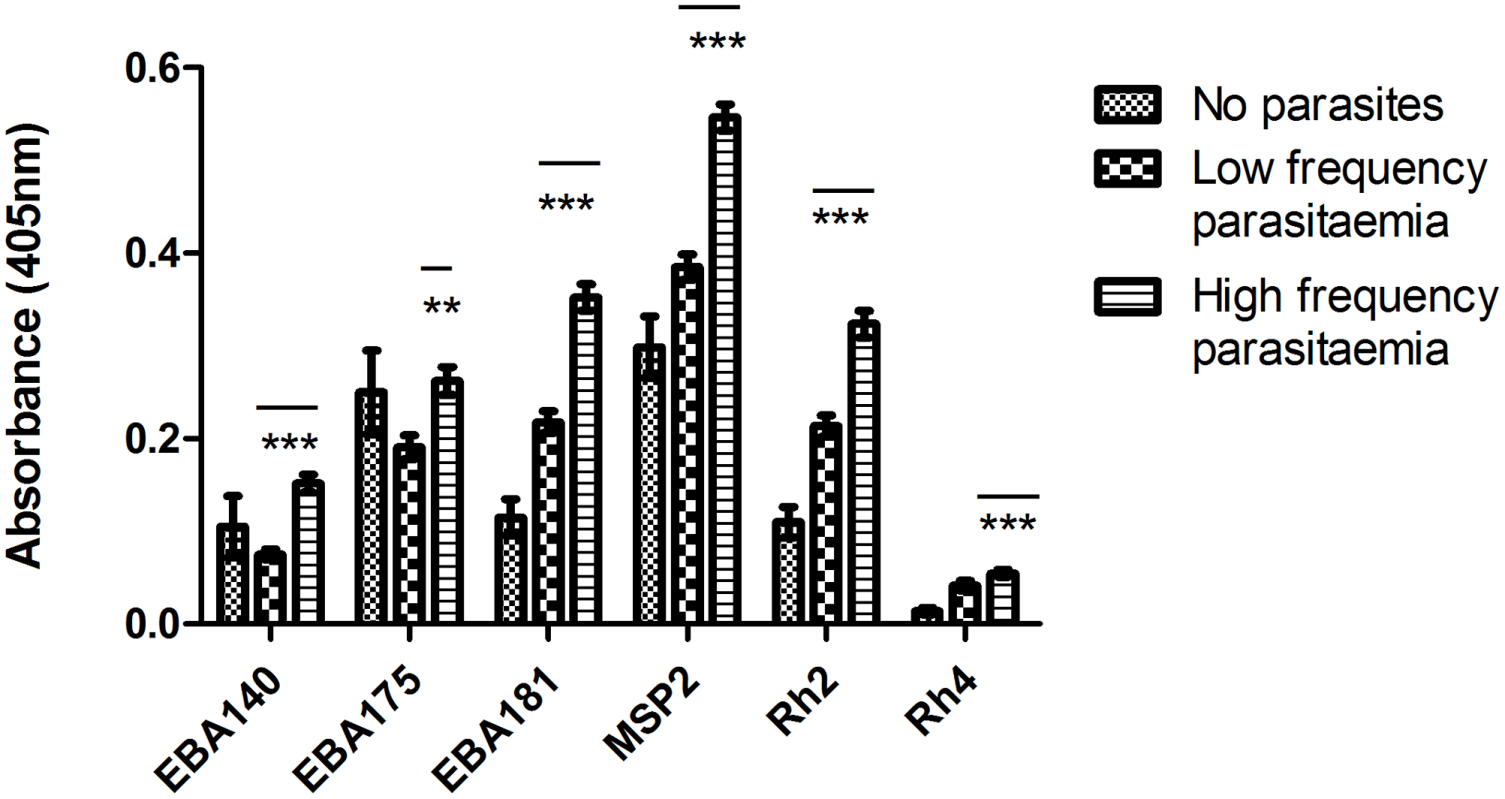
Comparison of mean antibody levels between individuals with varying frequency of parasitaemia. No parasites, n = 4 (32 samples); Low frequency parasitaemia, n = 67 (495 samples); and High frequency parasitaemia n = 85 (593 samples). Bars represent the mean antibody levels and SEM (***, p<0.0001; **, p<0.01).

In order to better understand antibody responses to different EBA and PfRh invasion ligands, IgG antibody levels to EBA140, EBA175, EBA181, PfRh2, PfRh4 were measured in plasma samples longitudinally collected from 156 individuals. We also evaluated antibodies to MSP2 as a representative example of a merozoite surface protein that is not involved in phenotypic variation in invasion pathways, and is an established vaccine candidate [10]. Seroprevalence (detection at least once during the study period) of antibodies to EBA140, EBA175, EBA181, PfRh2, PfRh4 and MSP2 were 66%, 80%, 86%, 91%, 43% and 97%, respectively. IgG antibodies produced against all antigens were significantly higher (p<0.0001 for all, except EBA175 with p = 0.01) in individuals with high frequency of parasitaemia compared with individuals that seldom had parasitaemia (Fig 1). Individuals in which no parasites were detected had, in general, the lowest antibody levels for most antigens. However, there were no statistical differences between antibody levels for those with no parasites compared to those that had low frequency parasitaemia.

**Fig 2:**
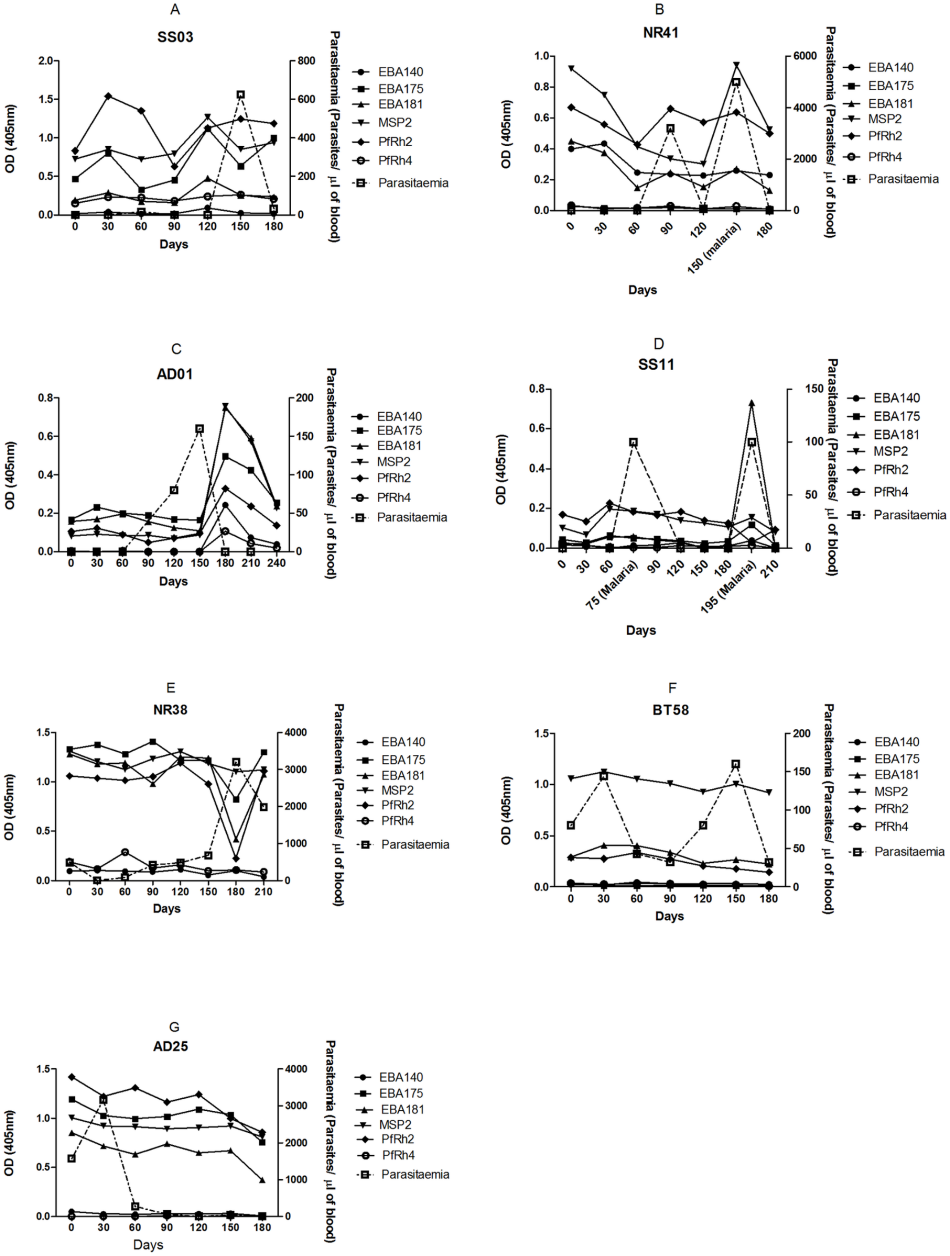
Longitudinal antibody profile showing changes in antibody levels measured by ELISA and breadth of antibody responses in representative individuals over time. The scales of the y-axes on all the graphs are different due to the uniqueness of the titre values and parasitaemia of each individual. Individuals (2B and 2D) with ‘‘malaria” on some days had clinical symptoms of malaria on those occasions.

Individuals varied in their antibody response profiles over time with respect to antibody levels and antigen specificity (Fig 2A and 2B). Sudden spikes (Fig 2C and 2D) or depressions (Fig 2E) in antibody levels to some or even all the antigens tested in this work were observed in certain individuals. The change in antibody level in some of the individuals coincided with drastic increase in parasitaemia (Fig 2E) or development of clinical symptoms as seen in individual SS11 (Fig 2D) who selectively showed a very large increase in EBA181 antibodies (and a slight increase in EBA175 antibodies) on day 195 at the second episode of malaria. The selectivity in antibody response became more conspicuous in some individuals; for example participant BT58 consistently produced high IgG antibodies to MSP2 only (Fig 2F); whereas EBA175, EBA181, MSP2, and PfRh2 antibodies were selectively elevated overtime in AD25 (Fig 2G).

More individual antibody profiles are provided in the supporting information (S2 Fig and S1 Table). When the number of individuals who showed consistently high antibody levels (>75^th^ percentile) was investigated, it was found that the number of antigens to which high antibody levels were produced tended to increase more with age (p<0.001) than frequency of exposure to *P*. *falciparum* parasites (p = 0.02).

Unlike the variability in antibody levels observed at the level of each individual participant, the overall means of the OD antibody levels to all antigens followed a general pattern through the seasons: elevated during the rainy season but lowered through the dry season (statistically significant for EBA175, p = 0.02; PfRh4, p = 0.02; EBA181, p = 0.02; and MSP2, p = 0.03).

Clustering analysis using agglomerative hierarchical clustering method showed that individuals that constantly produced high antibody levels to EBA181 also had the propensity to produce high antibody levels to PfRh2 (S3 Fig). Those that produced high antibody levels to EBA140 were also likely to produce high antibody levels to EBA175.

### Longevity of antibodies produced against the panel of antigens

**Fig 3:**
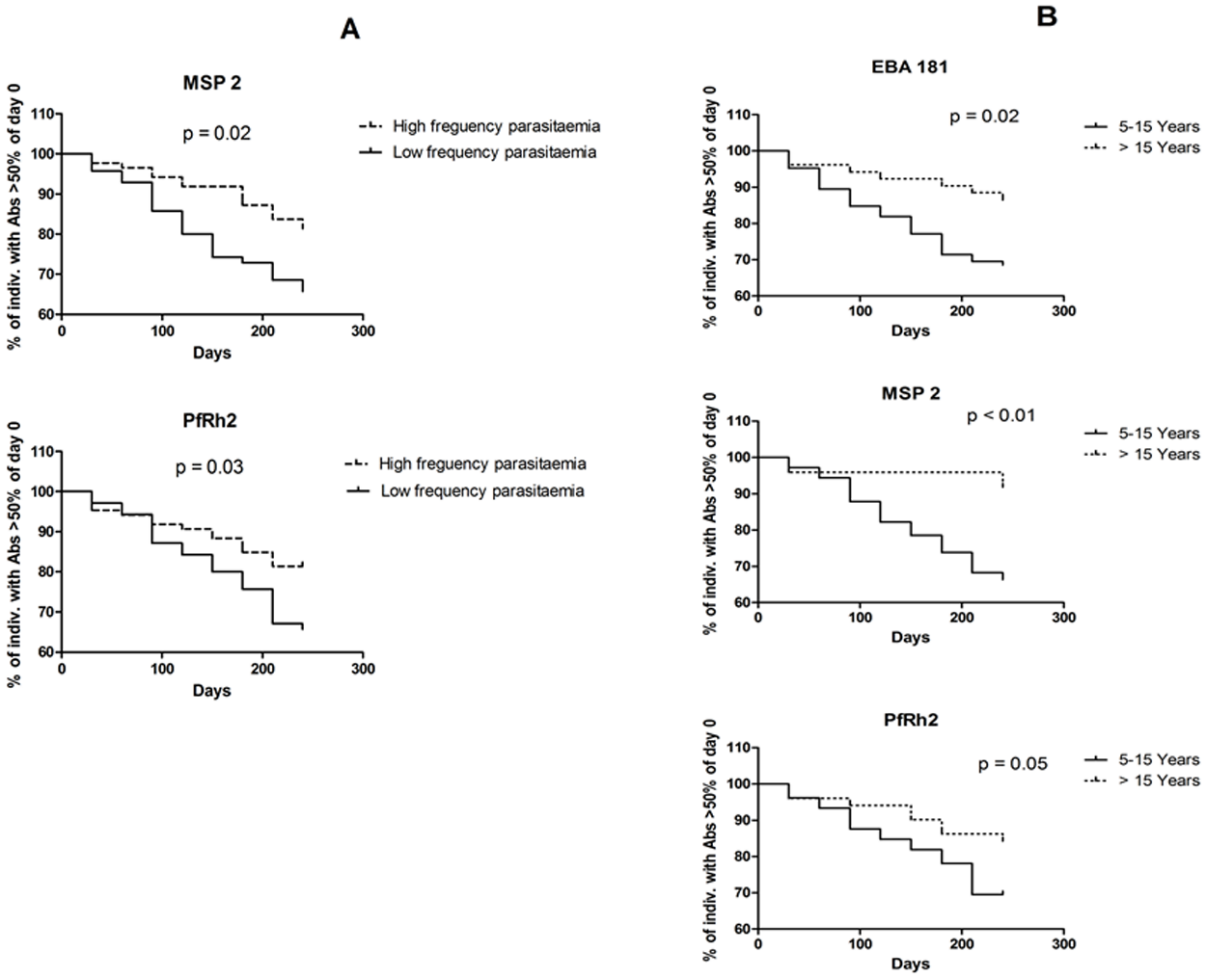
Kaplan- Meier survival curves showing the proportion of individuals with antibody levels of >50% of the day 0 antibody level for all antigens among the different age groups and among individuals with different levels of exposure to *P*. *falciparum*.

The effects of age and frequency of exposure to *P*. *falciparum* on the rate of decline of antibodies produced against the different antigens were determined by measuring the time-point at which the antibody level produced against each antigen reached 50% or less of the antibody level of day 0 (the first sample collection date) in the age and exposure groups. The survival analyses showed no significant differences in the decay of antibody to most of the antigens between individuals with low or high frequency of exposure to *P*. *falciparum* malaria parasites, except for antibody responses to PfRh2 (p = 0.03) and MSP2 (p = 0.02) which lasted longer in individuals that had more frequent *P*. *falciparum* infections (Fig 3A).

Similar comparisons between antibody responses in younger (below 15 years) and older individuals (≥15 years) were also carried out to determine the effect of age on antibody longevity. EBA175 (p = 0.98), EBA140 (p = 0.18) and PfRh4 (p = 0.77) antibody longevities were not affected by age. Antibodies to other antigens degenerated more rapidly in younger individuals (EBA181, p = 0.02; PfRh2, p = 0.05) and the decline of MSP2 antibodies in younger individuals was highly significant (p = 0.001) (Fig 3B). In conclusion, the results of ELISA-measured longitudinal IgG antibody response have shown that the regions of EBAs, PfRh2 and MSP2 used in this study are highly immunogenic in the Nigerian population studied, albeit with extensive variation in inter-individual and within-individual responses over time.

### EBA-specific invasion inhibitory antibodies

In order to gain further insights into the functional roles of EBAs in invasion inhibition, 3D7 parasite lines in which the EBAs had been genetically disrupted and their parental lines were used to test the inhibitory activities of the longitudinal plasma samples collected monthly from the participants. More inhibition of the parental line compared with a line that lacks a particular EBA protein in the presence of plasma antibodies, could be attributed to the presence of antibodies that were directed to the particular EBA protein [14]. This may point to the roles of the EBA protein in immune evasion or in determining the antigenic properties of the parasite. Conversely, more inhibition of any EBA knockout parasite relative to the parental line may indicate the presence of antibodies directed towards PfRhs proteins [28,40].

**Fig 4:**
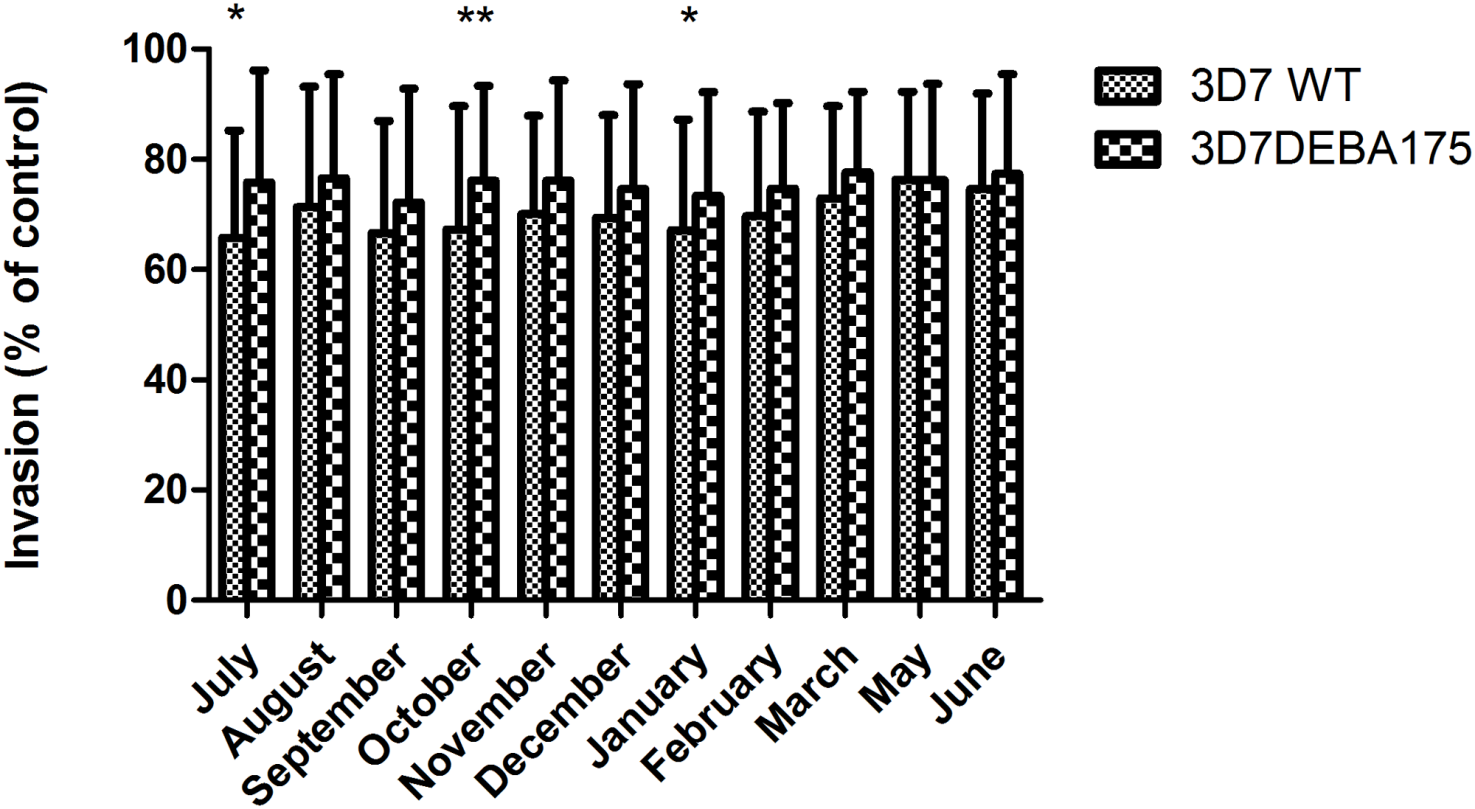
Seasonal variation in overall differential inhibition of 3D7 WT and the knockout lines. There were no differences between the overall inhibition between the 3D7 WT and EBA knockout parasites through the seasons except for EBA175 knockout parasites in the months of July, October, and January. Values represent mean of all samples ± SEM.

Unlike other EBA knockout parasites, 3D7 WT had significant overall difference in inhibition only compared with 3D7ΔEBA175 and only in the months of July (p<0.05), October (p<0.01) and January (p<0.05) (Fig 4).

**Fig 5:**
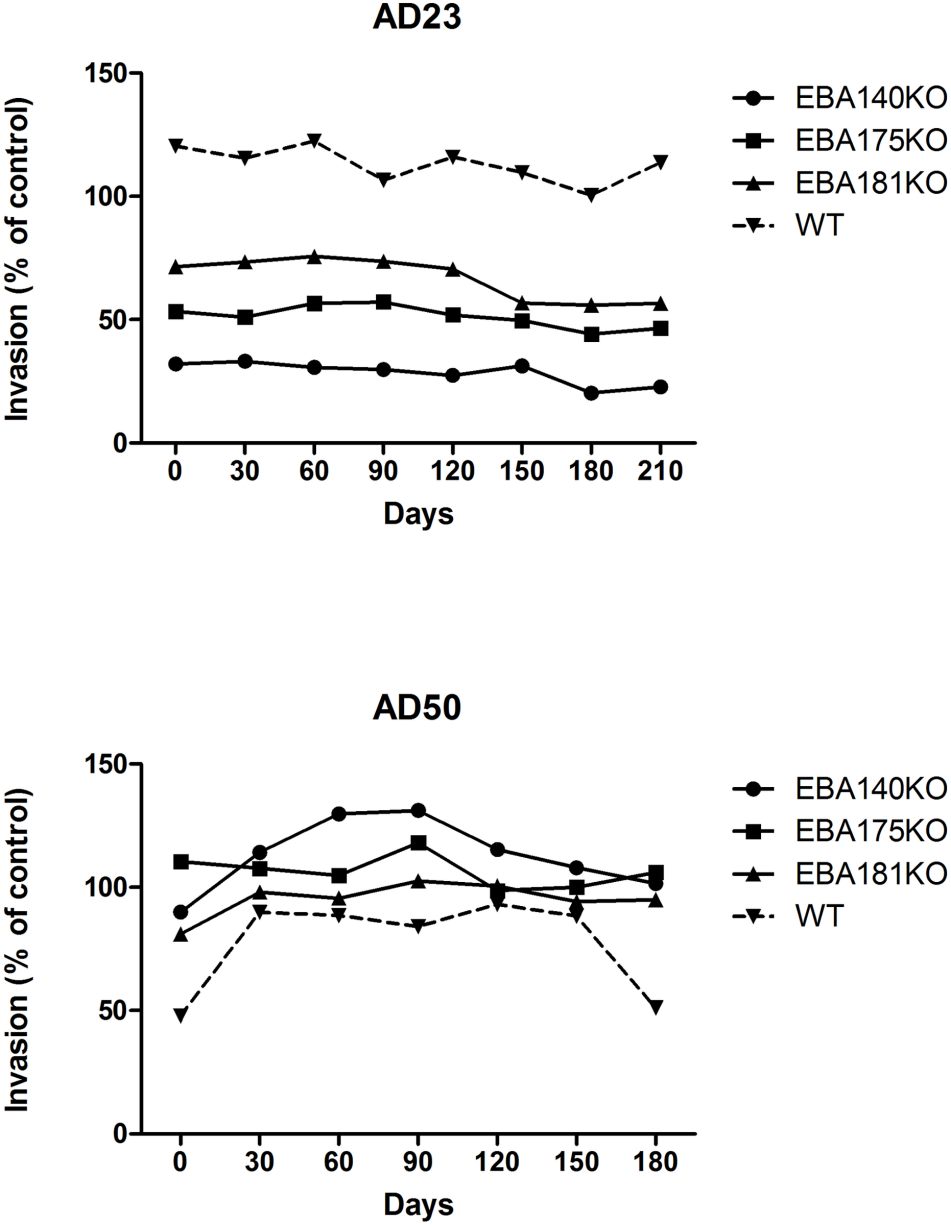
Longitudinal invasion inhibitory activity profiles of representative individuals. Individuals exhibited diverse invasion inhibitory activity against the 3D7 WT relative to the EBA knockout parasites overtime. Individual AD23 inhibited the EBA knockout parasites more than the parental parasite; individual AD50 inhibited the parental parasite more than the EBA knockout parasite lines.

Individuals show very diverse patterns in the differential inhibition over time when comparing the inhibition of the 3D7WT line with the knockout lines (Fig 5).

**Fig 6:**
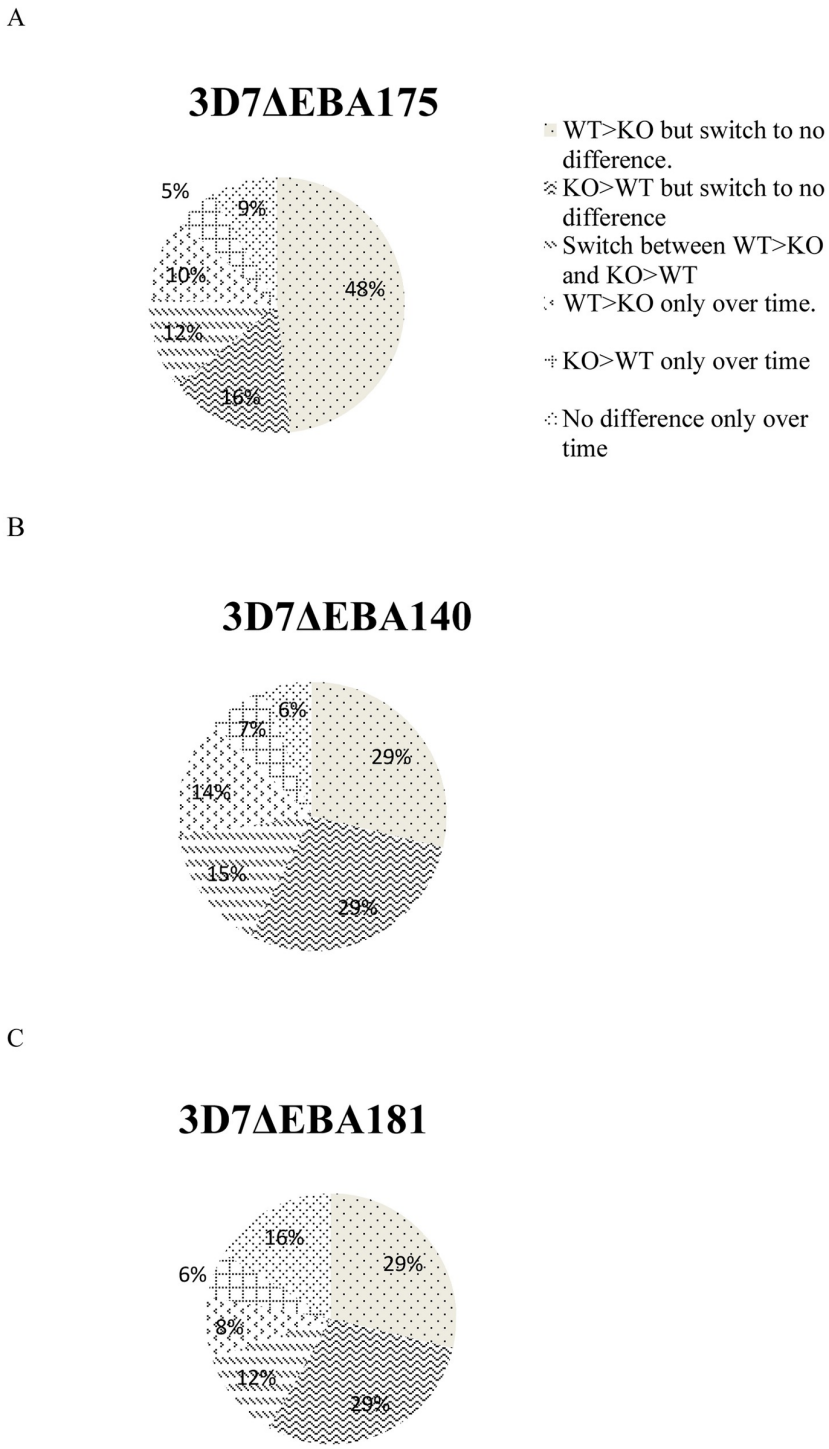
Pattern of differential inhibition of *P*. *falciparum* lines exhibited by individuals over time. The pie charts show the proportion of individuals that differentially inhibited 3D7WT invasion compared with 3D7 lines with genetically disrupted EBA genes (KO). The proportions of individuals with all observed outcomes are represented with various shades as shown. For example, “KO>WT only over time” means that the knock-out parasite was inhibited more than the wild-type and this was a stable pattern over time in the studied individuals.

More individual invasion inhibitory activity profiles are provided in the supporting information (S4 Fig and S1 Table). A high proportion of the participants, 70%, showed inhibition of the parental parasite more than 3D7ΔEBA175 at least once during the study. Importantly, the inhibition of the parental line more than 3D7ΔEBA175 was not a fixed phenotype of these individuals as 12% switched to responses that inhibited 3D7ΔEBA175 more than 3D7WT and 48% intermittently switched to responses that produced no differential inhibition of the parasite lines (Fig 6A). 16% switched between inhibiting 3D7ΔEBA175 more than 3D7WT and showing no difference between the lines. In total, 76% of the participants showed a pattern that switched between different inhibition patterns while only 24% showed a stable pattern (Fig 6A).

For both EBA140 and EBA181, the pattern was similar to that seen for EBA175; a majority of individuals switched between responses over time (73% for EBA140 and 70% for EBA181), and smaller groups had a stable pattern (27% for EBA140 and 30% for EBA181) as shown in Fig 6.

These findings show that inhibitory antibodies were directed against all three of the investigated EBAs, with predominance for antibodies directed against EBA175. Conversely, inhibitory antibodies that target ligands that mediated invasion as a result of the disruption of the different EBAs, shown by inhibition of knockout parasites more than 3D7 WT, were also produced by the study participants, albeit less frequently (Fig 6). In conclusion, the above results showed that EBAs elicit invasion inhibitory antibodies that may either be stable, or switch to other targets over time.

### Inhibitory antibodies targeting invasion pathways and parasitaemia

**Fig 7:**
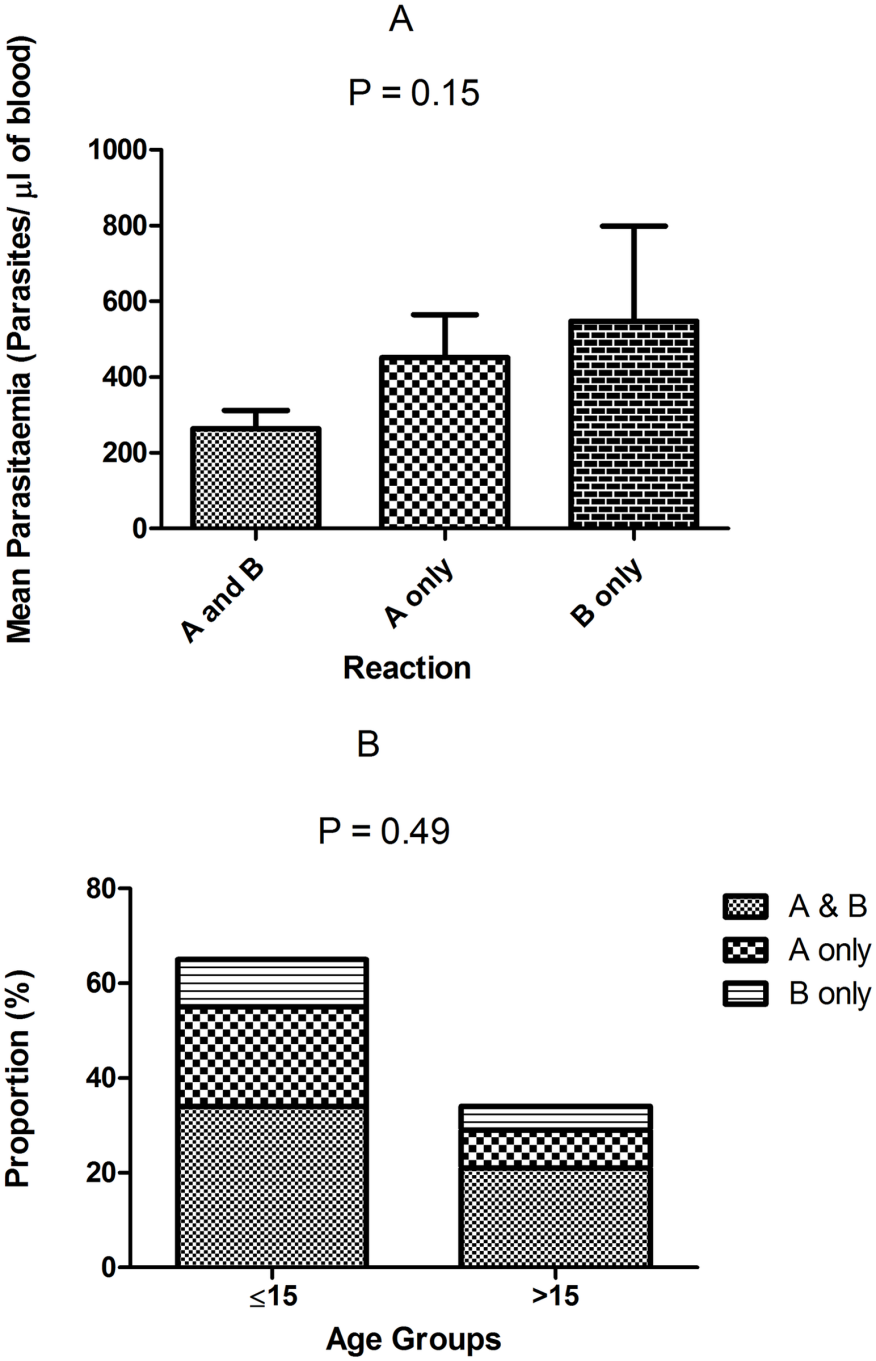
Relationship between invasion inhibitory antibodies to invasion pathway ligands parasitaemia and age. (A) Effects of antibodies directed against SA-dependent or SA-independent pathways on the mean parasitaemia. “**A**”, “**B**” represent individuals with only antibody responses to SA-dependent or SA-independent pathway invasion ligands, respectively. “**A and B”** represents individuals that presented a response to both pathways. (B) Effect of age on the acquisition of antibody responses to the two invasion pathways.

We also attempted to understand the possible protective roles of antibodies produced against ligands of SA- dependent and SA-independent invasion pathways. The 3D7 parasites are known to invade via SA-dependent pathways (using EBA140, EBA175 and EBA181) and SA-independent pathways relying on PfRh2, PfRh4 and other yet to be discovered ligands. Since the inhibition of 3D7WT more than any of the knockout parasites point to the presence of inhibitory antibodies directed against ligands that mediate SA-dependent pathways and the inhibition of knockout parasites more than the 3D7WT suggest the presence of inhibitory antibodies to SA-independent ligands [28], all the study participants were categorized into one of three groups: (i) those that produced only inhibitory antibodies that inhibited 3D7WT more than any of the knockout parasite throughout the study period, termed category A; (ii) individuals that had only inhibitory antibodies that inhibited the knockout parasites more than the 3D7WT i.e. inhibitory antibodies to SA-independent pathway, termed category B; (iii) individuals that had both responses i.e. those that produced inhibitory antibodies to SA-dependent pathway at one or more time points and also inhibitory antibodies that inhibit SA-independent pathway at one more time points. Individuals that produced inhibitory antibodies to both pathways overtime had the lowest mean parasitaemia compared with individuals that had inhibitory antibodies to only SA-dependent or SA-independent ligands, although the difference in mean parasitaemia between the groups was not statistically significant (p = 0.15) (Fig 7A). Furthermore, age did not have any effects on the types of response exhibited by the individuals (Fig 7B).

### Association between IgG antibody levels and invasion inhibition

**Fig 8:**
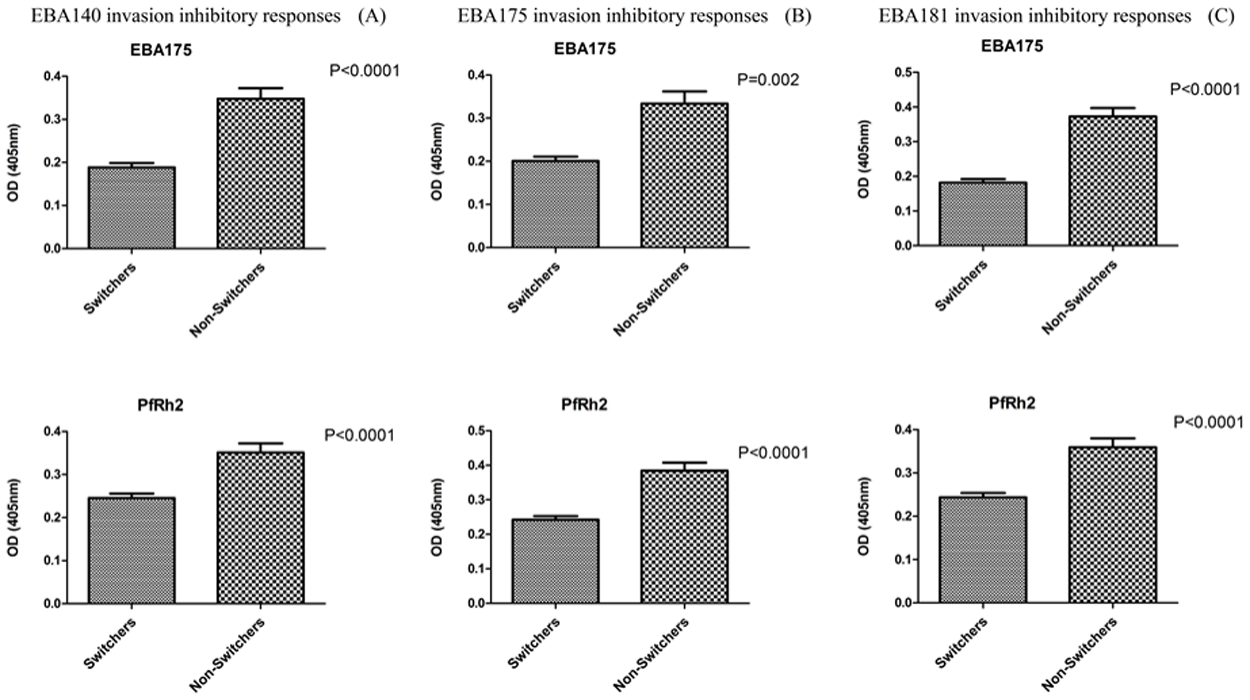
Comparison of EBA175 and PfRh2 antibody levels between individuals that switched between invasion inhibition patterns and those with unchanging invasion patterns. Bars represent the mean antibody levels and SEM. ‘‘Switchers” represents individuals that had fluctuating invasion inhibitory patterns while ‘‘Non-switchers” represents those that had stable invasion inhibitory pattern.

**Fig 9:**
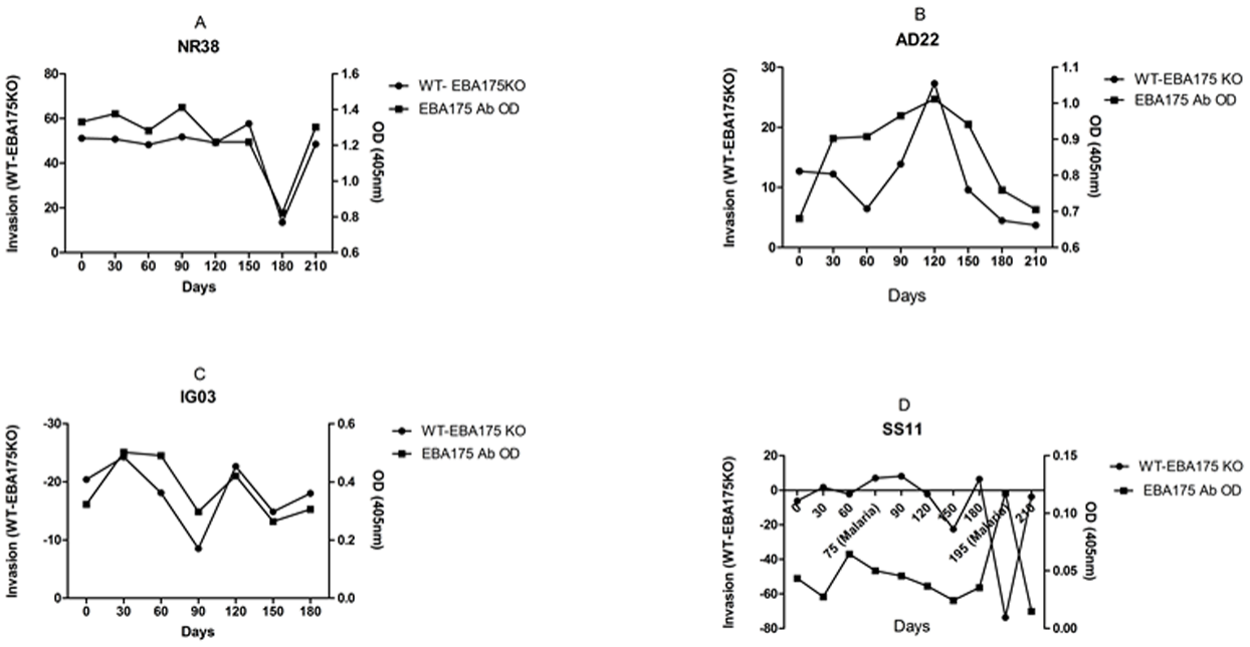
Correlation between EBA175 antibodies measured by ELISA and differential inhibition of 3D7WT and 3D7ΔEBA175 in four representative individuals. The scales of the y-axes on all the graphs are different due to individual differences in OD and percentage invasion values.

In each case of the EBA knockout lines, individuals that had a stable invasion inhibition pattern (“non-switchers”) throughout the study period produced significantly higher antibody levels (for all antigens tested) than individuals that switched between patterns, except for EBA140 and PfRh4 antibody levels in invasion inhibitory patterns of EBA140 and EBA175 knockout parasites, respectively (Fig 8A, 8B and 8C also S5A, S5B and S5C Fig). The longitudinal design of this study also provided an avenue to gain more insight into the nature of possible connection between IgG antibodies measured by ELISA and differential invasion inhibitory activities for the knockout lines, especially at the level of individual participants. For example, participants NR38 and AD22 produced EBA175 IgG antibodies in ELISA that positively correlated (NR38: r = 0.5, AD22: r = 0.57) with invasion results, indicating that when antibodies directed against EBA175 go down in levels in ELISA, they also play a less important role in invasion inhibition and other antibodies such as against PfRhs might be more important (Fig 9A and 9B). Participant IG03 had EBA175 IgG antibodies in ELISA that also correlated (r = 0.86) with invasion inhibition results, but here the decrease in antibodies in ELISA correlated more directly with a decrease in level of invasion inhibitory antibodies directed against EBA175 (Fig 9C).

One of the advantages of repeated sampling in immunoepidemiological studies was demonstrated in individual SS11 who produced EBA175 invasion inhibitory antibodies that coincided with increased EBA175 antibody production as measured by ELISA only on day 195 when he had malaria (Fig 9D), but had a poor overall correlation (r = 0.09).

### Association between haemoglobin type and invasion inhibition patterns

46 individuals out of all the study participants had HbAS blood genotype. For EBA140 (p = 0.01) and EBA180 (p = 0.006) invasion inhibitory responses, most of the individuals with the HbAS genotype (40 out of 46) switched between different invasion patterns over time. Individuals with the HbAA genotype did not produce any significant effect on the pattern of invasion inhibitory responses exhibited by the study participants to any of the EBAs. There were no significant differences in antibody levels produced against the majority of antigens studied between individuals with HbAA and HbAS, except for PfRh4 antibodies that were higher in HbAA individuals (p = 0.006).

## Discussion

*P*. *falciparum* has developed a strong capacity for immune evasion enabling repeated infections over time. An important mechanism contributing to immune evasion is the ability of *P*. *falciparum* to vary the expression and utilization of the EBA and PfRh invasion ligand families to evade acquired inhibitory antibodies. However, knowledge on the acquisition of functional antibody responses targeting these ligands has been limited. The majority of immuno-epidemiological studies conducted so far in endemic areas have been cross-sectional in design, with samples collected and antibodies tested at only one (or very few) time points [41]. In this study we evaluated antibodies to the EBA and PfRh ligands at multiple time points over almost a year to provide further evidence to understand the roles of EBAs and PfRhs as targets of immunity and immune evasion. We found that acquired invasion inhibitory antibodies to the EBAs may either be stable, or switch to other targets over time, suggesting that the immune response may be adapting to the diversity of invasion phenotypes to which it is exposed. We found that most individuals were ‘‘switchers” in response patterns, and only a minority show stable responses over time. Individuals that had a stable invasion inhibition pattern throughout the study period had significantly higher antibody levels overall than individuals that switched between patterns of invasion-inhibitory activity. Furthermore, there were broader and higher levels of antibodies to the EBA and PfRh ligands measured by ELISA in those with active parasitaemia or with greater exposure, suggesting that increasing exposure may contribute to the acquisition of antibodies of many specifies to different invasion phenotypes.

Most of the individuals, semi-immune children and immune adults, in this study had *P*. *falciparum* infection detected by microscopy at least once during the study period. This ability of malaria parasites to continuously re-infect or coexist with an activated immune response is not surprising considering the various immune evasion mechanisms deployable by malaria parasites in the skin, liver, blood and even in the mosquito stages [10,42]. Of note here is the possibility that children that had parasite-negative slides throughout the study period might have acquired the capability to control *P*. *falciparum* infections to levels below microscopic thresholds, as has been found in some studies before [43,44]. Importantly, the scenario observed in which infection intensity (mean parasitaemia) was slightly higher in the rainy season and lower in the dry season, but with a common occurrence of parasites detectable in the blood throughout the seasons, may suggest that the upsurge in malaria cases or parasite intensity usually recorded in the rainy seasons is due to transmission of antigenically distinct malaria parasites between individuals. Indeed, a recent study in a west African country has shown that the number of clonal infections per individual could range from 1 to 8, depending on other characteristics of an area [45]. The sustained parasite prevalence through the seasons shown here is also in agreement with previous results obtained from our study site that showed 42% and 48% prevalence for the dry and rainy seasons, respectively [46].

IgG antibody responses measured by ELISA showed that the panel of antigens tested in this study are targets of antibodies and the seroprevalence recorded is comparable with those that have been demonstrated in other populations [36,37,47]. However, PfRh4 was not widely recognized in our study population and this may suggest that the circulating parasites in this area do not express (or express low levels of) PfRh4. Parasite isolates from neighbouring West African countries have recently been shown to express low levels of PfRh4 compared with other EBA and PfRh proteins [13]. Comparatively, MSP2 appears to be more immunogenic protein and this may be due to its location on the merozoite and its abundance [48]. EBA140 and EBA175 may be playing more important roles in protection against high level parasitaemia since their levels were slightly higher (although not significant) in the few individuals that presented parasite-negative slides throughout the study period. These two proteins have also been found by [5] to be highly protective against clinical malaria compared to a number of other merozoite proteins.

The longitudinal design of this work offered the opportunity to understand the nature and development of antibody responses from an individual’s perspective. We found that the levels of IgG antibodies and choice of antigen to which these antibodies were produced varied markedly within and between individuals over time. The variation observed may represent differences in an individual’s immunological experience, genetics and age. Prior studies have also reported that there can be substantial fluctuation in antimalarial antibody levels over time [49,50]. This knowledge of how antibodies are acquired and maintained over time may help inform the development of long-lasting vaccines and serological tools for malaria surveillance. The tendency for some individuals to continuously respond to selected antigens while ignoring others is reminiscent of a paradox of immune response called original antigenic sin, or clonal imprinting [51]. In this circumstance, an individual will continue to produce a high antibody response to an antigenic variant of the parasite/pathogen that first primed his or her immune response even in the presence of a new antigenic challenge. The existence of original antigenic sin in malaria immunity was first suggested by [52] and later by [53] while studying antibody responses to MSP2, MSP1 and Pf260/230 in The Gambia. A confirmation of this concept in immune responses to merozoite antigens was demonstrated recently [54] by comparing antibody responses in sequential and mixed immunization protocols in rabbits. If clonal imprinting is a valid explanation for our observation here, then, it carries very important implications for the deployment of malaria vaccines in endemic areas, where most children would have been naturally exposed to various malarial antigens before immunization. Future improvements in adjuvant/vaccine technology may help eliminate this bottleneck as has been demonstrated for influenza vaccines [55].

The breadth of antibody responses in individuals that continuously produce high antibody responses was more influenced by age than persistent parasites in the peripheral circulation. This is in agreement with earlier works that have found an important role for age in determining the breadth of antibody response to some merozoite antigens [56].

Another observation from this current study is the tendency of individuals that consistently produced high levels of antibodies to EBA181 to also mount a high response against PfRh2, and then EBA140 and EBA175 antibody responses also appeared to be linked. An earlier study has suggested that EBA181 and PfRh2 may be functionally related [40] and it has been shown that Senegalese parasite isolates that expressed high levels of *eba181* also had high levels of *PfRh*2, while Ghanaian isolates with low *eba181* levels had low levels of *PfRh2* [13]. The rarity of individuals that continuously produced high antibody levels to all antigens tested in this study could be a demonstration of a high level of evolutionary success for malaria parasites, in suppressing a powerful immune system that has the ability to generate over 10 billion different antibodies [57].

In our study there were antigen-specific differences in the longevity of IgG antibodies, as MSP2 antibodies declined more rapidly in younger individuals and needed more frequent parasite encounters to be maintained in plasma. This is in accordance with an earlier study [58] and it has been suggested that this rapid decline of MSP2 antibodies may be attributed to the stimulation of short-lived B cells by repeat sequences that are commonly found in MSP2. In contrast to MSP2 antibodies, the longevities of EBA140, EBA175 and PfRh4 antibodies were the same in both adults and children. Unlike the other EBAs and PfRh4, EBA181 and PfRh2 antibodies degenerated more rapidly in younger children and PfRh2 antibodies also required high frequency parasitaemias. These differences in antibody longevity might be a reflection of subtle structural differences between these antigens, or perhaps a parasite controlled factor in the presentation of antigens to the immune system.

One of the important results from our study is the propensity of many individuals to show clear switching of invasion inhibitory capacity for different antigens over time. The genetic disruption of individual EBAs clearly changed the antigenic properties of the parasites as seen by differential inhibition of the modified compared to the parental 3D7 parasites. This indicates that varied expression of EBAs may help parasites evade the immune system and this agrees with previous cross-sectional studies using the same parasite strains in a Kenyan population [14,28]. Unlike what was observed in the Kenyan population where invasion inhibitory antibodies to EBA140 were the most prevalent, more individuals produced antibodies that inhibited 3D7WT more than 3D7ΔEBA175 in this current study, an indication that inhibitory antibodies that target EBA175 are more widely produced in our study population. This is followed by inhibitory antibodies to EBA140 and then EBA181. This order of prevalence is in agreement with recent results that showed that parasite isolates from some West African countries produced higher levels of *eba175* followed by *eba140* and then *eba181* transcripts [13]. The fluctuating invasion inhibition pattern observed in individuals over time may help explain the reasons why it has been difficult to identify any immune correlate of protection for most antigens or vaccines.

The prevalence pattern of invasion inhibitory antibodies we observed is slightly different from the one obtained using ELISA and could simply be because live parasites used in *in vitro* inhibition presented EBAs in their native and correctly folded forms, and therefore measured antibodies to more epitopes than ELISA that detected antibodies to only RIII-V. In essence, the invasion inhibitory antibodies against EBA175 may represent those targeting RII, RIII-V as well as other regions. A limitation of our study is the number of samples included, however, we believe that since there is so little longitudinal data of this kind in the literature, the results are of value and can supply important information to the field of stability of antibodies in malaria.

A recently developed functional binding assay has revealed that inhibitory antibodies produced against EBA175 were mainly targeting the EBA175 RII region [59]. However, RII has been shown to be highly polymorphic and under diversifying selection with as many as 32 alleles with several non-synonymous substitutions in Thailand alone [60]. Of importance in the current study is the existence of individuals that switched from responses that inhibited 3D7WT more than the 3D7 knockout parasites to responses that inhibited 3D7 knockout parasites more than 3D7WT or even responses that produce no differential inhibition of the parasite lines. These changes in the targets of invasion inhibitory activities might represent attempts of individuals to mount antibody responses to parasites of different invasion phenotypes that they encounter over time. Studies in comparable populations in West Africa have demonstrated the co-existence of parasites of different invasion phenotypes in the same population [13,61]. Higher antibody levels observed in individuals that maintained a stable response pattern over time may imply that acquisition of high antibody levels against a particular invasion phenotype may persist for some time irrespective of the phenotypes of newly invading parasites. Further studies may be required to determine whether switching or having a stable response pattern is more advantageous to the host, but it could be speculated that since high antibody levels were associated with a stable response, this should imply a higher level of immunity. It is also interesting to note that most of the individuals with HbAS genotype were switchers. If HbAS is able to restrict erythrocyte invasion as suggested by [62] then HbAS may be able to select for parasites that can invade through alternative ligands in a manner similar to the selection of PfEMP1 variants as proposed by [63] and hence the possession of antibodies produced against different invasion ligands.

Our findings here suggest that the conflicting observations that have been reported in earlier studies about the correlation between ELISA measured IgG and invasion inhibition might be related to unique immune characteristics of individuals. This is because the longitudinal samples from some individuals showed an almost perfect correlation between IgG measured by ELISA and invasion inhibitory antibodies while no, weak, or negative correlations were observed in others. Then the analyses of cross-sectional samples for each month of sampling obscured the correlations that were observed at the individual level. This again points to the importance of longitudinal studies involving multiple sampling for understanding the basics of immune response against malaria.

In summary, our results support the conclusion that selective expression of EBAs and PfRhs is an important mechanism for immune evasion. The nature of the invasion inhibitory antibodies varied heavily within and between individuals over time in most individuals, something that could have been concealed if only a cross-sectional bleed had been performed. Our data contribute to the understanding of naturally acquired immune responses to malaria.

## Supporting information

S1 Fig Variation in mean parasitaemia and parasite prevalence over time. Mean monthly parasitaemia (bars) vary through the season, proportion of individuals that were infected at any particular time point (parasite prevalence, indicated with lines) did not change significantly through the seasons. Rainy season months: April-October, 2009; dry season months: November-March, 2009/2010. There was no sampling in the month of April due to logistic challenges caused by false rumours about this work circulated by uninformed locals.
(TIF)

S2 Fig Longitudinal antibody profiles of more individuals showing changes in antibody levels over time. (TIF)

S3 Fig A dendogram showing clusters of individuals obtained by hierarchical clustering of their consistent production of high IgG antibody (>75^th^ percentiles) to the panel of antigens. Values 0–25 represent the rescaled distance between the different clusters. The higher the rescaled distance, the higher the dissimilarity between the clusters. EBA181 and PfRh2 formed a cluster with the shortest rescaled distance, followed by EBA140 and EBA175. Other clusters have rescaled distance very close to 25.
(TIF)

S4 Fig Longitudinal invasion inhibitory activity profiles of more representative individuals. (TIF)

S5 Fig Comparison of mean antibody levels between individuals that had stable invasion inhibition pattern and those that switched between patterns over time. (TIF)

S1 Table Invasion and ELISA (OD) data for all individuals included in this study. The OD values were all measure at 405nM and percent invasion were determined relative to the control.
(XLSX)
