# Supplementary figures and images for: Acquisition, maintenance and adaptation of invasion inhibitory antibodies against Plasmodium falciparum invasion ligands involved in immune evasion
Source: PLoS One. 2017 Aug 7;12(8):e0182187. doi: 10.1371/journal.pone.0182187 (PMC5546579; doi:10.1371/journal.pone.0182187)

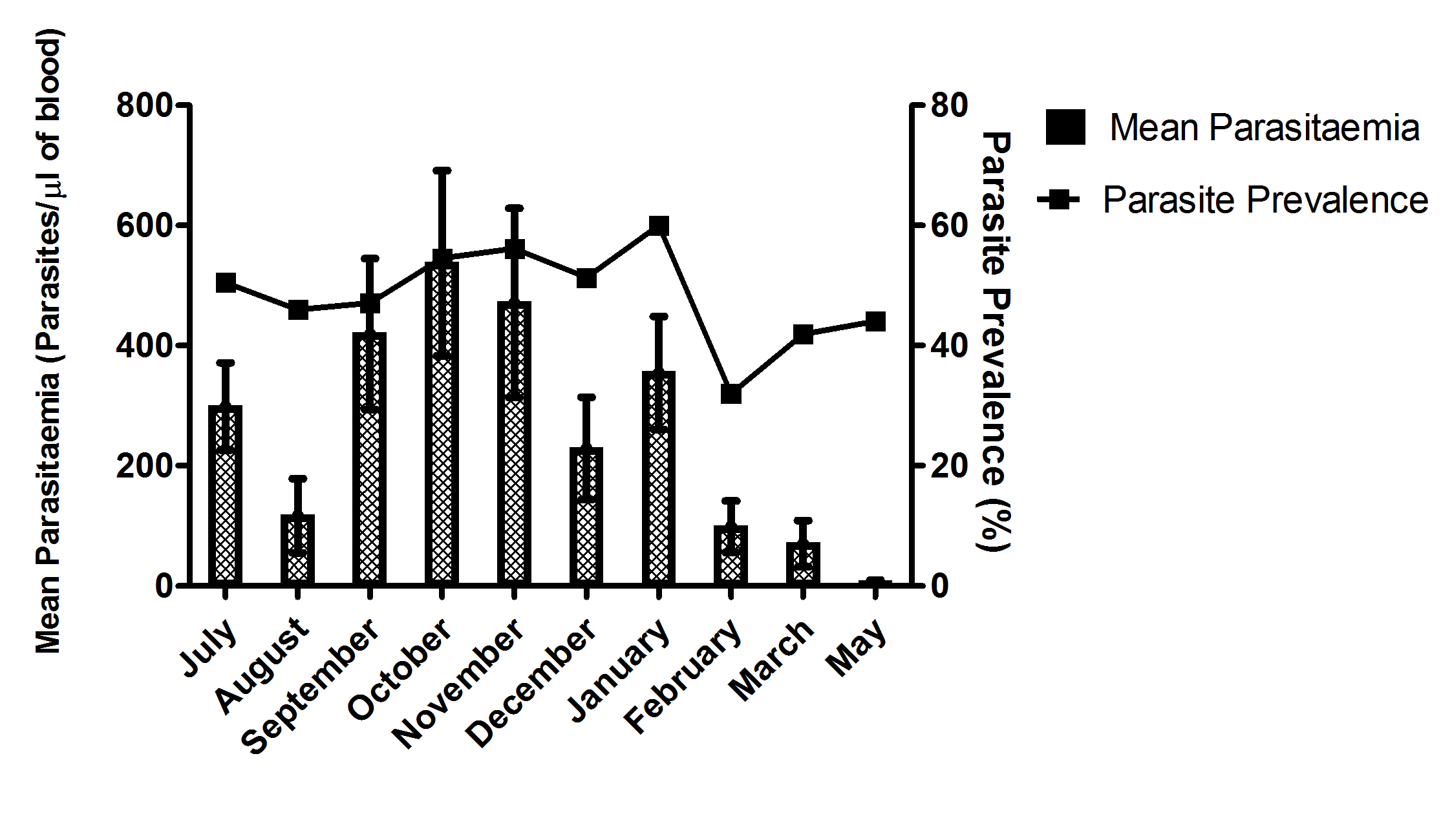

Supplement: S1 Fig — Mean monthly parasitaemia (bars) vary through the season, proportion of individuals that were infected at any particular time point (parasite prevalence, indicated with lines) did not change significantly through the seasons. Rainy season months: April-October, 2009; dry season months: November-March, 2009/2010. There was no sampling in the month of April due to logistic challenges caused by false rumours about this work circulated by uninformed locals. (TIF) [file pone.0182187.s001.tif]

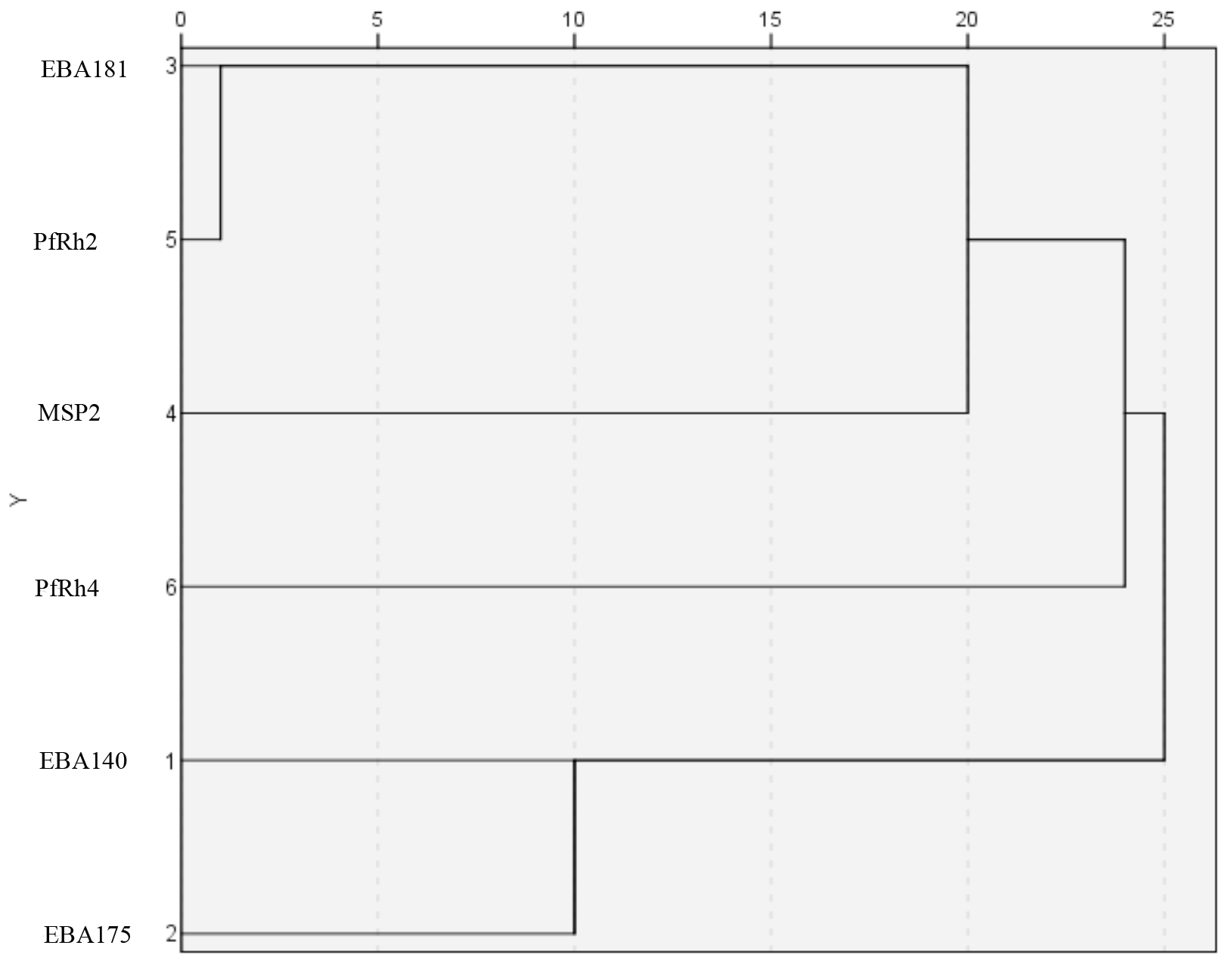

Supplement: S3 Fig — Values 0–25 represent the rescaled distance between the different clusters. The higher the rescaled distance, the higher the dissimilarity between the clusters. EBA181 and PfRh2 formed a cluster with the shortest rescaled distance, followed by EBA140 and EBA175. Other clusters have rescaled distance very close to 25. (TIF) [file pone.0182187.s003.tif]

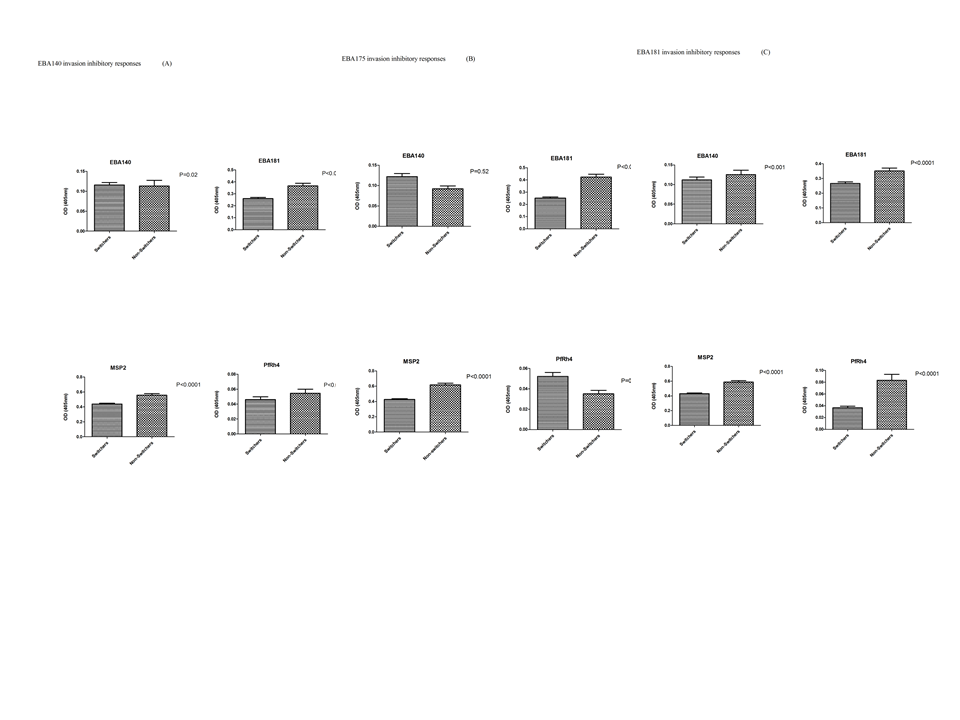

Supplement: S5 Fig — (TIF) [file pone.0182187.s005.tif]
